# Comparison of Short-Term Versus Long-Term Antibiotic Therapy Among Severe Cases of Pneumonia: A Prospective Observational Study Among Children

**DOI:** 10.7759/cureus.35298

**Published:** 2023-02-22

**Authors:** Dnyaneshwar Potpalle, Sandeep Gada, Amar Devaguru, Narendra Behera, Mummareddi Dinesh Eshwar

**Affiliations:** 1 Paediatrics, Mahavir Institute of Medical Sciences, Vikarabad, IND; 2 Paediatrics, Peoples Hospital, Hyderabad, IND; 3 Paediatrics, MKCG (Maharaja Krushna Chandra Gajapati) Medical College And Hospital, Berhampur, IND

**Keywords:** dyspnea, acute lower respiratory infection, india, long-term vs short term, antibiotic therapy, children, community-acquired pneumonia, pneumonia

## Abstract

Introduction

Pneumonia continues to be the leading cause of morbidity and mortality in children younger than five years. The World Health Organization (WHO) recommends intravenous antibiotics for five days for severe pneumonia. However, the optimum duration of parenteral antibiotic therapy for pneumonia is not practicable and feasible in poor and resource-constrained settings like India. Given the current Indian scenario wherein childhood pneumonia is extremely prevalent, we attempted to undertake this study to compare the duration of antibiotic therapy in severe cases of community-acquired pneumonia (CAP).

Methods

A prospective observational study was carried out on 225 cases of severe and very severe CAP patients at a tertiary care center. The study group included children between two months to five years of age. The participants were subjected to antibiotic therapy (parenteral) plus supportive care. The selection of antibiotics was empirical and according to the WHO acute respiratory infection control program. Hematological parameters including blood hemoglobin, C-reactive protein, erythrocyte sedimentation rate (ESR), and total leukocyte count, and radiological evaluation were performed on all the participants. Cases were followed up for the duration of clinical response.

Results

Out of the 225 cases, 25 patients did not respond to antibiotics and were categorized as the treatment failure group. Of the remaining 200 cases, 104 (52%) showed clinical response within three days (3.0±0.016), and 96 showed a response in four to seven days (4.4±0.064). The mean duration of antibiotic therapy among short-course versus long-course treatment was statistically significant (p<0.0001). The majority of patients developed leukocytosis, neutrophilia, and elevated ESR.

Conclusion

Short-course parenteral antibiotics therapy was equally effective as long-course therapy in severe pneumonia. However, very severe pneumonia patients required a longer course of parenteral antibiotics therapy. Very severe pneumonia was significantly associated with high mortality and treatment failure.

## Introduction

Pneumonia is an acute lower respiratory infection that affects lung parenchyma. Pneumonia continues to be the leading cause of morbidity and mortality in children younger than five years. Globally, pneumonia accounts for the largest number of childhood deaths each year in this age group. According to World Health Organization (WHO), pneumonia contributes to around 20% of deaths among children under five years with excessive mortality being reported from South Asia and sub-Saharan Africa [1]. Moreover, 90% of all pneumonia cases in children occur in low- and middle-income countries [2]. A high risk of death due to poor access to medication and healthcare facilities along with financial and infrastructural limitations in low-income countries is a cause for serious concern [3].

As per the WHO acute respiratory infection (ARI) control program, the treatment and management for severe and very severe community-acquired pneumonia (CAP) in children under five years is hospitalization and administration of parenteral antibiotics [4].

Currently, there are few randomized control trials that highlighted the benefit of short-course antibiotic therapy in non-severe pneumonia [5,6]. However, there are scanty studies that evaluated the effectiveness of short-course parenteral antibiotic therapy against severe and very severe CAP in children under five years of age [7,8]. Many often optimum duration of parenteral antibiotic therapy for pneumonia is not practicable and feasible in poor and resource-limited settings such as India [9].

Keeping the view of morbidity and mortality patterns of the Indian scenario during childhood, we undertake this study to establish the optimal duration of clinical response to antibiotic therapy and to compare the clinical effectiveness of short-term versus long-term antibiotic therapy in CAP among children aged between two months and five years.

## Materials and methods

This prospective observational study was carried out in the department of pediatrics attached to Maharaja Krushna Chandra Gajapati (MKCG) Medical College and Hospital, Berhampur, Odisha, India. The study period was two years (September 2013-August 2015). The study protocol was approved by the Institutional Ethics Committee of MKCG Medical College, Brahmapur, Odisha, India (approval number: IEC/188). Prior informed consent was obtained from the parents/guardians of the study participants. All the consecutive patients diagnosed with severe pneumonia and very severe pneumonia and admitted to the hospital were enrolled in this study as per pre-defined inclusion and exclusion criteria.

Hematological parameters including blood hemoglobin, C-reactive protein (CRP), erythrocyte sedimentation rate (ESR), total leukocyte count, and radiological evaluation were performed on all the participants. Cases were followed up for the duration of clinical response.

Inclusion and exclusion criteria

Patients aged between two months and five years and diagnosed with severe pneumonia showing lower chest indrawing with or without tachypnea, and very severe pneumonia patients who presented with cough or difficulty in breathing/dyspnea, with signs and symptoms of pneumonia and any one of the accompanying critical signs like cyanosis, inability to drink, stridor in calm child, and lethargy were included in the study.

All patients with hospital-acquired pneumonia, children with human immunodeficiency virus (HIV) infection, heart diseases, recurrent wheezing with chest indrawing, sepsis, meningitis, severe acute malnutrition, immunosuppressive and other syndromic conditions, and those who showed remission in one to two days were excluded from the study.

The study groups included were subjected to parenteral antibiotic therapy along with supportive care as recommended by the WHO ARI control program recommendations [10]. The details of antibiotic treatment among the study participants are shown in Table 1.

**Table 1:** Parenteral antibiotic therapy given to the study participants

Therapy	Choice of first-line antibiotics				Added antibiotics	Other antibiotics
Antibiotics given	Ampicillin	Gentamicin	Ceftriaxone	Amikacin	Cloxacillin	Vancomycin /linezolid /piperacillin+tazobactam
Dose	50 mg/kg/ dose/4 doses	2.5 mg/kg/dose/2 doses	50mg/kg/ dose/2 doses	7.5 mg/kg/dose/2 doses	50mg/kg/dose/4 doses	As per dose

The patients who showed clinical response were discharged with an oral antibiotic prescription. Those children who failed to respond to first-line antibiotics within 48 hours, received second-line antibiotics (cloxacillin, ceftriaxone, ampicillin). Cloxacillin was considered in the case of empyema/massive consolidation. All children were evaluated during the hospital stay and the response to treatment was noted. Those who were showing clinical improvement by 48 hours continued the same antibiotics till the clinical response was achieved. Clinical response is defined as the resolution of baseline symptoms and clinical signs related to pneumonia without the need for additional or alternative antibiotics therapy. The cases that responded to intravenous antibiotics therapy within three days were included under short-course and those who took more than three days (four to seven days) to respond were considered long-course.

Statistical analysis

Continuous data were represented as mean ± standard deviation. Categorical data were expressed as numbers in percentage. The student t-test was used to determine significant differences between the two groups for parametric data. The odds ratio was determined whenever required. The significance of the statistical tests was predetermined at a probability value of 0.05 or less (p<0.05). The data were analyzed by using the statistical software Graphpad Prism version 5 (2007/08; Dotmatics, Boston, Massachusetts, United States).

## Results

Of the total 225 severe cases of CAP included in the study majority were within the age group of 2-12 months and showed male preponderance (68%) compared to females (38%) and the male-to-female ratio was 2.12:1. The age group and sex-wise distribution of the study subjects are shown in Table 2.

**Table 2:** Age group and sex-wise distribution of study participants

Age in months	Female, n (%)	Male, n (%)	Total, n (%)
2-12	59 (34%)	114 (66%)	173 (76%)
13-60	14 (26%)	38 (74%)	52 (24%)
Total	73 (32%)	152 (68%)	225 (100%)

Among the cases included, the majority (84.44%) presented with severe pneumonia compared to very severe pneumonia (15.55%). Cough (100%), fever (92.4%), and hurried breathing (100%) were the most common symptoms noted among the study participants. The details of the presenting symptoms among the cases included are given in Table 3.

**Table 3:** The presenting symptoms among the study participants (n=225)

Symptoms	n (%)
Cough	225 (100)
Fever	208 (92.44)
Hurried breathing	225 (100)
Refusal of feeds	28 (12.44)
Stridor in calm child	01 (0.45)
Convulsions	08 (3.63)
Cyanosis	06 (2.72)
Lethargy	28 (12.44)

Chest retractions and tachypnea were present in all cases (100%), crepitations were heard in 71.11%, and wheezing in 44.44% of patients. Other signs included bronchial breathing (17.77%), diminished breath sounds (4.44%), and grunting heard in 13.33% of cases.

Routine blood investigations showed leucocytosis (51%), neutrophilia (56%), and lymphocytosis (15.11%). ESR was elevated in 62.66% of cases and anemia was observed in 49% of cases. The details of the hematological abnormalities among the study participants are depicted in Table 4.

**Table 4:** Hematological abnormalities noted among the study participants (n=225) Hb: Hemoglobin; TLC: Total leukocyte count; ESR: Erythrocyte sedimentation rate; SD: Standard deviation

Hematological abnormality	n (%)	Mean±SD
Anemia (Hb<11gm%)	110 (48.88)	10.85±1.79
Leucocytosis (TLC>15000/mm^3^)	116 (51.55)	11130.45±4694.8
Neutrophilia (>60%)	126 (56)	53.52±8.11
Lymphocytosis (>35%)	34 (15.11)	28.76±3.13
Elevated ESR (>20mm/hr)	141 (62.66)	23.30±13.89

Among the 225 participants included in the study, 115 (51%) showed radiological findings of pneumonia. The majority revealed consolidation (43.37%) and interstitial pneumonia (34.78%). Other presentations included empyema (5.21%), pleural effusion (1.73%), and collapse (1.73%) all leading to complications (8.69%). The detailed radiological data is presented in Table 5.

**Table 5:** Radiological findings among the study participants (n=225)

Radiological findings	No. of cases, n (%)
Pneumonic consolidation	50 (43.47)
Bronchopneumonia	15 (13.04)
Empyema	6 (5.21)
Pleural effusion	2 (1.73)
Collapse	2 (1.73)
Interstitial pneumonia	40 (34.78)
Total	115 (100)

A majority (62.22%) of the patients were prescribed ampicillin+gentamicin/amikacin (62.22%) as the first-line antibiotic treatment. Furthermore, some (37.77%) patients were prescribed ceftrixone+amikacin/gentamicin. Other antibiotics used among the participants included cloxacillin, ampicillin, ceftriaxone, vancomycin, and linezolid. The details of the antibiotic prescription among the study participants are shown in Table 6.

**Table 6:** Antibiotic prescription among the study participants (n=225)

Antibiotics	n (%)
Ampicillin+gentamicin/amikacin	140 (62.22)
Ceftrixone+amikacin/ gentamicin	85 (37.77)
Cloxacillin	10 (4.44)
Ampicillin	02 (01)
Ceftriaxone	4 (1.77)
Vancomycin	4 (1.77)
Linezolid	1 (0.4)

Out of 200 cases, 104 (52%) have shown clinical response within three days (short course), and 96 have shown response between four to seven days (long course). The mean duration among the short-course participants was 3.0±0.016 and among long-course treatment participants, it was 4.4±0.064. Student t-test revealed that there was a significant difference between short-course and long-course duration of treatment (p< 0.0001), which means short-course parenteral antibiotics therapy was found to be equally effective as long-course parenteral antibiotics therapy as depicted in Table 7.

**Table 7:** A comparison of treatment response with short-course and long-course therapy *: Statistically significant SE: Standard error; t: Ratio of the difference between the mean of the two sample sets and the variation that exists within the sample sets; Df: Degrees of freedom; p: Probability value/level of significance

Clinical response	Duration (days)	Mean±SE (days)	Df	‘t’ value	p- value
Respiratory rate below the cut-off point, absence of chest indrawing, absence of any danger signs, and clinical improvement in symptoms	<3 (n=104)	3.0± 0.016	198	22	<0.0001*
Respiratory rate below the cut-off point, absence of chest indrawing, absence of any danger signs, and clinical improvement in symptoms.	4-7 (n=96)	4.4±0.064			

The severity of pneumonia was significantly associated with the duration of clinical response. Very severe pneumonia required a longer course of parenteral antibiotic therapy in comparison to severe pneumonia (P=0.013). The patient's age (p=1) and sex (p=0.9468) were not found to be associated with treatment response. There was no significant difference (0.7972) between the two commonly used treatment regimens as shown in Table 8.

**Table 8:** Comparison of treatment responses with reference to age, sex, and severity of pneumonia *: Statistically significant

Parameter	Variable	Short-course	Long-course	p-value
Age	<12 months	78	72	1
	>12 months	26	24	
Sex	Male	70	70	0.9468
	Female	34	36	
Pneumonia	Severe	99	76	0.013*
	Very severe	5	20	
Antibiotics	Ampicillin+gentamicin/amikacin	70	62	0.7972
	Ceftrixone+amikacin/ gentamicin	34	34	

Most of the patients (88.88%) were cured with first-line antibiotics and discharged with an oral antibiotic prescription. However, 10 (4.44%) patients developed complications during the treatment. Nine (4%) patients died during treatment, and 25 (11.11%) patients did not respond to the treatment. Assessment of risk factors for mortality showed that very severe pneumonia was significantly associated with mortality as compared to severe pneumonia (p<0.001) as shown in Table 9.

**Table 9:** Risk factors for mortality *: Statistically significant

Parameter	Variable	No. of patients who did not survive (n=9)	No. of patients who survived (n=216)	p-value	Odds ratio
Age	<12 months	07	166	0.9485	1.054
	>12 months	02	50		
Sex	Male	06	148	0.9386	0.9459
	Female	03	70		
Pneumonia	Severe	02	188	<0.001*	0.04255*
	Very severe	07	28		

The assessment of treatment failure revealed that nonresponse at 48 hours was significantly associated with predictors like very severe pneumonia (p<0.0001) and complications (p<0.001). However, the age (p=0.3861), sex (p=0.7819), choice of antibiotic treatment (p=0.6794), and blood oxygen saturation (p=0.8499) were not significantly associated with treatment failure as shown in Table 10.

**Table 10:** Predictors of treatment failure among the study participants *: Statistically significant SpO2: Oxygen saturation

Parameter	Variable	Treatment failure	Successful	p-value
Age	<12 months	17	156	0.3861
	>12 months	8	44	
Sex	Mal	18	134	0.7819
	Female	7	66	
Antibiotics	Ampicillin+gentamicin/amikacin	17	123	0.6794
	Ceftrixone+amikacin/ gentamicin	8	77	
Pneumonia	Severe	13	177	<0.0001*
	Very severe	12	23	
Complication	Yes	10	0	<0.001*
	No	15	200	
SpO2	<90	14	108	0.8499
	>90	11	92	

## Discussion

There are several recommendations and guidelines available across the globe for the treatment and management of pneumonia [11]. The management of pneumonia is performed based on the severity, age, and presence of co-morbidities among other factors [12]. Parenteral antibiotic therapy is recommended for the treatment of the severe form of pneumonia. Moreover, the duration of treatment may depend on the severity of pneumonia and other clinical characteristic features. Given the emergence of antibiotic resistance among bacteria that cause pneumonia, it is prudent to choose the antibiotic and the duration of therapy carefully [13]. According to the guidelines recommended by the WHO ARI control program, CAP in children is classified as severe pneumonia among those who present tachypnea and recessions, and very severe pneumonia when patients show additional symptoms like hypoxemia, dullness, or inability to drink. The choice and duration of antibiotics may vary based on the severity of pneumonia, the causative microbe, prevailing antimicrobial susceptibility patterns, and other characteristic features. 

There is a debate over the choice and effectiveness of short-course and long-course antibiotic therapy in children with CAP. It was observed that five days of antibiotic therapy may be sufficient among children with uncomplicated pneumonia that does not require hospitalization [6]. Interestingly, another study has found that a three-day short course of antibiotic therapy was equally efficient as a seven-day course in the treatment of childhood pneumonia [14]. 

Moreover, the short-course antibiotic therapy among non-hospitalized CAP patients was not inferior to standard 10-day long-course therapy. Such short-course therapy may minimize the adverse drug effects, and the possibility of antimicrobial resistance, and lower the economic burden [15].

This study's results showed that short-course parenteral antibiotics therapy is equally effective as long-course therapy. The mean duration among short-course treatment was 3.0±0.016 days and among long-course treatment, it was 4.4±0.064 days. The severity of pneumonia was significantly associated with the duration of clinical response. Very severe pneumonia required a longer course of parenteral antibiotics therapy. The early initiation of short-course intravenous antibiotic treatment followed by a transition to an oral antibiotic was found to be substantially less expensive and has comparable efficacy to conventional intravenous treatment for seven days [16].

In our study, most patients (88.88%) were cured with first-line antibiotics and discharged with a prescription for oral antibiotics. About 4.4% of cases developed complications during treatment and 4% of cases died during treatment. A total of 25 (11.11%) cases exhibited treatment failure that included complicated cases and deaths. These results corroborated with the previous study carried out by Mishra et al. in Indian settings [17]. 

The case fatality rate (CFR) was 4% in our study, which differed from some previous studies that reported varied case fatality rates ranging from 3.4% to 12,8% [17-19]. Underlying congenital heart disease (CHD) was a significant risk factor for pneumonia mortality in some previous studies. The exclusion of pneumonia associated with CHD may be the probable reason for the low CFR seen in our study.

It was found that very severe pneumonia was significantly associated with mortality. Some previous studies have reported that age of less than one year, CHD, very severe pneumonia, and malnutrition were significant predictors of mortality [18,20,21]. In the present study, patients with CHD and malnutrition were excluded.

Study limitations

The major limitation of our study is the study design that excluded children with comorbidities. This is not a randomized control trial and, therefore, the results may be influenced by bias. Another limitation of our study is a single-center analysis, which may not be representative of the entire population. Moreover, this study does not present information on the etiology of pneumonia and the results of the antimicrobial susceptibility profile of microbes responsible for pneumonia among the cases.

## Conclusions

Since pneumonia causes significant morbidity and accounts for mortality among children below five years of age, the management of this condition assumes increased significance. Moreover, pneumonia increasingly affects people living in economically poor and developing nations who are plagued with financial constraints and limited access to healthcare. Therefore, it is essential to evaluate the treatment modalities in the management of patients with pneumonia. Given the increased prevalence of antimicrobial resistance, the choice and duration of antibiotic therapy also require careful consideration. In this study, we noted that short-course parenteral antibiotic therapy was equally effective as a long-course treatment in severe pneumonia patients. However, very severe pneumonia patients required a longer course of parenteral antibiotics therapy, which was also significantly associated with high mortality and treatment failure.

## References

[1] (2023). WHO: Pneumonia in children. https://www.who.int/news-room/fact-sheets/detail/pneumonia.

[2] Marangu D, Zar HJ (2019). Childhood pneumonia in low-and-middle-income countries: an update. Paediatr Respir Rev.

[3] Sheikh M, Jehan F (2021). Using big data for risk stratification of childhood pneumonia in low-income and middle-income countries (LMICs): Challenges and opportunities. EBioMedicine.

[4] (1984). A programme for controlling acute respiratory infections in children: memorandum from a WHO Meeting. Bull World Health Organ.

[5] Li Q, Zhou Q, Florez ID (2022). Short-course vs long-course antibiotic therapy for children with nonsevere community-acquired pneumonia: a systematic review and meta-analysis. JAMA Pediatr.

[6] Pernica JM, Harman S, Kam AJ (2021). Short-course antimicrobial therapy for pediatric community-acquired pneumonia: the SAFER randomized clinical trial. JAMA Pediatr.

[7] Saul H, Gursul D, Cassidy S, Bielicki J (2022). A short course of antibiotics is effective for treating pneumonia in children. BMJ.

[8] Khilnani G, Zirpe K, Hadda V (2019). Guidelines for antibiotic prescription in intensive care unit. Indian J Crit Care Med.

[9] Gupta S, Lodha R, Kabra SK (2018). Antimicrobial therapy in community-acquired pneumonia in children. Curr Infect Dis Rep.

[10] (2023). Acute respiratory infections in children : case management in small hospitals in developing countries, a manual for doctors and other senior health workers. Acute Respiratory Infections In Children : Case Management In Small Hospitals In Developing Countries, A Manual For Doctors And Other Senior Health Workers.

[11] Chou CC, Shen CF, Chen SJ (2019). Recommendations and guidelines for the treatment of pneumonia in Taiwan. J Microbiol Immunol Infect.

[12] Gupta D, Agarwal R, Aggarwal AN (2012). Guidelines for diagnosis and management of community- and hospital-acquired pneumonia in adults: Joint ICS/NCCP(I) recommendations. Lung India.

[13] Grimwood K, Fong SM, Ooi MH, Nathan AM, Chang AB (2016). Antibiotics in childhood pneumonia: how long is long enough?. Pneumonia (Nathan).

[14] Bielicki JA, Stöhr W, Barratt S (2021). Effect of amoxicillin dose and treatment duration on the need for antibiotic re-treatment in children with community-acquired pneumonia: the CAP-IT randomized clinical trial. JAMA.

[15] Williams DJ, Creech CB, Walter EB (2022). Short- vs standard-course outpatient antibiotic therapy for community-acquired pneumonia in children: the SCOUT-CAP randomized clinical trial. JAMA Pediatr.

[16] Omidvari K, de Boisblanc BP, Karam G, Nelson S, Haponik E, Summer W (1998). Early transition to oral antibiotic therapy for community-acquired pneumonia: duration of therapy, clinical outcomes, and cost analysis. Respir Med.

[17] Mishra S, Kumar H, Anand VK, Patwari AK, Sharma D (1993). ARI control programme: results in hospitalized children. J Trop Pediatr.

[18] MacIntyre CR, McIntyre PB, Cagney M (2003). Community-based estimates of incidence and risk factors for childhood pneumonia in Western Sydney. Epidemiol Infect.

[19] Sehgal V, Sethi GR, Sachdev HP, Satyanarayana L (1997). Predictors of mortality in subjects hospitalized with acute lower respiratory tract infections. Indian Pediatr.

[20] Reddaiah VP, Kapoor SK (1995). Acute respiratory infections in underfives experience at comprehensive rural health services project hospital, Ballabgarh. Indian J Community Med.

[21] Deivanayagam N, Nedunchelian K, Ramasamy S, Sudhandirakannan Sudhandirakannan, Ratnam SR (1992). Risk factors for fatal pneumonia: a case control study. Indian Pediatr.

